# Test System Stability and Natural Variability of a *Lemna Gibba* L. Bioassay

**DOI:** 10.1371/journal.pone.0003133

**Published:** 2008-09-04

**Authors:** Claudia Scherr, Meinhard Simon, Jörg Spranger, Stephan Baumgartner

**Affiliations:** 1 Research Institute of Organic Agriculture, Frick, Switzerland; 2 Society for Cancer Research, Institute Hiscia, Arlesheim, Switzerland; 3 Institute for Chemistry and Biology of the Marine Environment ICBM, University of Oldenburg, Oldenburg, Germany; 4 Institute of Complementary Medicine KIKOM, University of Bern, Bern, Switzerland; Purdue University, United States of America

## Abstract

**Background:**

In ecotoxicological and environmental studies *Lemna* spp. are used as test organisms due to their small size, rapid predominantly vegetative reproduction, easy handling and high sensitivity to various chemicals. However, there is not much information available concerning spatial and temporal stability of experimental set-ups used for *Lemna* bioassays, though this is essential for interpretation and reliability of results. We therefore investigated stability and natural variability of a *Lemna gibba* bioassay assessing area-related and frond number-related growth rates under controlled laboratory conditions over about one year.

**Methology/Principal Findings:**

*Lemna gibba* L. was grown in beakers with Steinberg medium for one week. Area-related and frond number-related growth rates (r_(area)_ and r_(num)_) were determined with a non-destructive image processing system.

To assess inter-experimental stability, 35 independent experiments were performed with 10 beakers each in the course of one year. We observed changes in growth rates by a factor of two over time. These did not correlate well with temperature or relative humidity in the growth chamber.

In order to assess intra-experimental stability, we analysed six systematic negative control experiments (nontoxicant tests) with 96 replicate beakers each. Evaluation showed that the chosen experimental set-up was stable and did not produce false positive results. The coefficient of variation was lower for r_(area)_ (2.99%) than for r_(num)_ (4.27%).

**Conclusions/Significance:**

It is hypothesised that the variations in growth rates over time under controlled conditions are partly due to endogenic periodicities in *Lemna gibba*. The relevance of these variations for toxicity investigations should be investigated more closely. Area-related growth rate seems to be more precise as non-destructive calculation parameter than number-related growth rate. Furthermore, we propose two new validity criteria for *Lemna gibba* bioassays: variability of average specific and section-by-section segmented growth rate, complementary to average specific growth rate as the only validity criterion existing in guidelines for duckweed bioassays.

## Introduction

Members of the Lemnaceae family occur in standing and slowly flowing waters all over the world, except in arctic and antarctic regions [1]. These small monocotyledonous flowering plants are relevant to many aquatic ecosystems, providing food and habitat for various animals and microorganisms. Their morphological and physiological characteristics are well known since they have been intensively investigated (for reviews see [2]–[4]). Their small size and rapid, predominantly vegetative reproduction forming genetically uniform clones make them valuable research organisms for studies in plant physiology, genetics, ecology and environmental monitoring [5]–[11]. Because of their high sensitivity to organic and inorganic substances *Lemna* species are also used as test organisms for water quality assessments as well as for ecotoxicological studies regarding adverse effects of e.g. herbicides, pharmaceuticals and heavy metals on aquatic plants [12]–[19]. For testing water quality or testing of chemicals with the duckweed growth inhibition test the two species *Lemna minor* L. and *Lemna gibba* L. are used in national and international guidelines [20]–[22].

The methodological quality of a laboratory investigation depends amongst others on the uniformity of the experimental conditions as well as on the inclusion of a sufficient number of appropriate controls. Thus, close investigation of the entire experimental set-up prior to main experiments with test substances is recommended in guidelines for the *Lemna* bioassay [20], [21] to determine the acceptability of the materials used (e.g. glassware, growth medium etc.) and the handling procedures for the growth of the selected duckweed species. Nontoxicant tests (systematic negative control experiments), where all vessels contain only growth medium without any added test material, provide corresponding information, also concerning possible location effects in the growth chamber as well as the variability within or between replicates. In addition, for appropriate interpretation of the results from a set of experiments conducted over a longer time span, it is also essential to know the variability range of the experimental set-up over this period. Duckweeds, like all living organisms, may exhibit considerable variations in their growth and metabolic activity over time even under controlled laboratory conditions [23], [24]. Endogeneous rhythms have been described even on a molecular level [7].

Though most *Lemna* spp. tests were performed under controlled laboratory conditions, there is not much information available regarding the growth dynamics of untreated controls as well as the stability of the experimental set-up used over the time course of typical test periods. We therefore investigated stability and natural variability of a *Lemna gibba* bioassay under controlled laboratory conditions over about one year. *Lemna gibba* L. was grown in beakers with modified Steinberg medium (moStM) for one week. Frond area and frond number were measured with a commercial non-destructive image processing system at days 0, 3, 5, and 7 of every assay. Area-related and frond number-related growth rates (r_(area)_ and r_(num)_) were calculated from the data obtained.

Our first aim was to assess inter-experimental stability, i.e. to determine natural variations or possible rhythmic changes in duckweed growth over time. We therefore analysed 35 independent experiments with 10 beakers of untreated *Lemna gibba* control plants each, performed in the course of one year.

The second aim was to estimate intra-experimental stability. We thus analysed data of 96 test beakers of six full systematic negative control experiments (nontoxicant tests with pure moStM) each. The data were analysed in randomised groups of six beakers (‘pseudo-treatments’), since six replicates for the controls are recommended in guidelines for ecotoxicological tests with duckweeds [21], [22].

Furthermore, the variability of two calculation parameters, frond number-related growth rate (r_(num)_) and area-related growth rate (r_(area)_), were compared, frond number being the mandatory observation parameter in the guidelines mentioned above which must be combined with either frond area, dry weight, fresh weight or chlorophyll content as the second observation parameter.

## Materials and Methods

### Plants and general growth conditions

Duckweed, *Lemna gibba* L., was obtained from a laboratory culture of Aachen Technical University, Germany. Identity (clone no. 9352) was confirmed visually by E. Landolt (Department of Environmental Science, ETH Zurich) and genetically by K. Appenroth (Department of Plant Physiology, University of Jena).

Cultivation medium was modified Steinberg medium (moStM), prepared according to the draft ISO/DIS 20079 [25]. As suggested there, stock solutions 1 to 3, 8, and 9 were individually prepared with autoclaved distilled water (Büchi, Fontavapor 250, Flawil, Switzerland), while the stock solutions 4–7 were pooled. All bottles were wrapped in aluminium foil and kept in the refrigerator until use. Every week and prior to any experiments the final medium was freshly prepared with autoclaved distilled water and a pH of 5.9±0.1.

Duckweed cultures were grown in a plant growth chamber (180×75×100 cm, made of an aluminium frame with glass walls covered with white paper, constructed by technicians from the Research Institute of Organic Agriculture) illuminated with fluorescent lights (143±3 µmol photons m^−2^ s^−1^ PAR, TL-D 36W/33-640, Philips, Zürich, Switzerland). Deviating from guidelines, where continuous illumination is required [20]–[22], we used a light-to-dark period of 16 h : 8 h since this better reflects natural conditions of duckweed growth. Temperature and relative humidity (RH) were measured with a mechanical thermo- and hygrograph (Wilh. Lambrecht, Göttingen, Germany). The daily minima and maxima were extracted from the graphs and the mean values were calculated over seven days. During daytime the mean maximum temperature was 21.5±1°C and the mean maximum RH was 45±6%, and during night-time 17±1°C and 71±6%, respectively (mean±SD).

### Plant storage, adaptation and pre-culture

Long term storage (solid stock-cultures): For long term storage the plants were maintained aseptically as stock-cultures in 100 ml Erlenmeyer flasks containing 50 ml of solid moStM with 1% (w/v) Bacto® dextrose (Difco, Detroit, USA) and 1% (w/v) bacteriological agar No. 1 (Oxoid, Basingstoke, Great Britain). Dextrose was added in order to detect a possible bacterial contamination of the cultures. After two weeks in the growth chamber these cultures were stored at 7°C in the dark for about a month, before some of the duckweed colonies were transferred to freshly prepared solid medium.

Adaptation (liquid stock-cultures): The method of keeping solid stock-cultures in the dark requires a thorough adaptation of the plants to liquid medium which takes several weeks. Here, about eight colonies were transferred aseptically from the solid stock-cultures into 500 ml Erlenmeyer flasks containing 150 ml of autoclaved moStM. These liquid stock-cultures were then cultured under normal experimental conditions in a second identical growth chamber for a period of at least four weeks before further use and their medium was changed weekly.

Pre-culture: Afterwards, duckweeds from the liquid stock-cultures were grown in two glass vessels with 1.8 L of moStM each in the second identical growth chamber for three to four weeks prior to the experiments in order to obtain large numbers of plants. Young, rapidly growing colonies from these pre-cultures were put into similar glass vessels with freshly prepared medium every week, covering less than one third of the surface at the beginning of the week. It was ensured that rapid, near-exponential growth was maintained and was not restricted e.g. due to space limitation or limited nutrient availability.

### Main Experiments

On the day when an experiment began, test specimens with a bright green colour without visible lesions, chlorosis or necrosis were selected from one vessel. They were sorted according to number of fronds of similar size (e.g. three fronds per colony or three large and one small frond per colony, respectively) and were put into petri dishes with medium until use. If necessary, any stipules connecting daughter fronds to the pouch of the mother frond were carefully separated without injuring the fronds. These young and healthy plants were used as inoculum for all test beakers.

Stock solutions of moStM (50-fold concentrated) were mixed together immediately before use [25]. For every experiment 3.8 ml of the combined moStM stock solutions were pipetted into each beaker (100 ml, SIMAX®, Kavalier, Sázava, Czech Republic). Then 46.2 ml of autoclaved distilled water was added. Afterwards the sorted duckweed colonies were carefully put into the beakers at random, so that every beaker contained 10 fronds of similar size at the beginning of the experiment. Due to the restricted space in the growth chamber, we used 100 ml beakers to be able to increase the number of replicates in one experiment. Compared to ISO guidelines, we used lower volumes of moStM and fewer fronds (50 ml instead of 100 ml, and 10 instead of up to 16 fronds). However, it was assured that neither nutrient limitations nor overcrowding occurred during the 7 days of test duration.

All measurements of frond area and number were obtained with an image processing system (Scanalyzer, duckweed analytic software, version 3) [26]. For the recorded images frond number and frond area were determined automatically. Afterwards the quality of the automatic image analysis was checked for each image and corrected by hand if necessary.

After the initial measurement (day 0) each beaker was wrapped in black paper up to the surface of the test solution and put on black paper in the plant growth chamber in order to eliminate any diffused light from the side or the bottom. The light intensity at every location in the growth chamber had been measured previously. The beakers were placed in the growth chamber at places with similar light intensities at 5 cm distance to each other. Additionally, they were covered with watch-glasses (from the same manufacturing batch) to avoid excessive evaporation. Further measurements with the image processing system were taken on day 3, 5 and 7 of the experiment.

From the measured frond area and frond number the growth rate per day r [d^−1^] was calculated for the total test period (day 0–7; average specific growth rate), and for four other time intervals (day 0–3, 3–5, 3–7, 5–7; segmented growth rates) according to the equation:
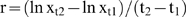
(1)where x_t1_ is the value of observation parameter at day t_1_, x_t2_ is the value of observation parameter at day t_2_, and t_2_−t_1_ is the time period between x_t1_ and x_t2_ in days. The parameters used for statistical analysis were area-related growth rates (r_(area)_) and frond number-related growth rates (r_(num)_).

### General experimental design

Annual variation in duckweed growth during 7 days was assessed in 35 experiments between January 2003 and January 2004. For this purpose untreated plants in 10 beakers with pure moStM were grown for one week, and frond area and frond number were assessed (see above). This study was part of a larger investigation on the effects of highly diluted substances on duckweed growth rate [27].

The stability of the entire experimental set-up in the growth chamber was investigated in six independent systematic negative control experiments with pure moStM (full-size experiments with 100 beakers) performed at different points in time during the year. Four beakers out of 100 were eliminated by a random procedure in order to obtain 16 groups of six beakers (96 beakers in total), since the guidelines for the duckweed growth inhibition test [21], [22] recommend at least six replicates for the controls. For every experiment each beaker was randomly placed within the growth chamber. However, in order to maintain constant physical conditions for each beaker the selected place remained the same within an experiment.

### Statistics

Data from the regular monitoring of duckweed growth rates were analysed using descriptive statistics and were illustrated graphically. Correlations with environmental parameters were calculated with nonparametric Spearman rank correlation.

From the systematic negative control experiments, data for a total of 576 beakers were obtained. Data from two beakers had to be excluded due to spilling. The experimental data were summarized using standard descriptive statistics. We calculated the variability of the average specific growth rates (day 0–7) for groups of six replicates as well as the variability of growth rate in time within one beaker. The latter was characterised by calculating the time-weighted section-by-section growth rate (day 0–3, 3–5 and 5–7) and the mean value of coefficients of variation (CV), according to the OECD guideline for freshwater algae and cyanobacteria growth inhibition test [28]. In addition, the data for r_(area)_ and r_(num)_ were evaluated for statistical significance based on analysis of variance (ANOVA) F tests, after checking the data for normal distribution with Shapiro-Wilk W Test and homogeneity of variance with Levene's Test. In a two-way analysis of variance the independent variables were experiment number and treatment (16 ‘pseudo-treatments’ with medium only) and the dependent variable was r_(area)_ or r_(num)_ (α = 5%), respectively. All analyses were carried out with the software STATISTICA version 6.0 (Stat Soft, Inc., Tulsa, OK, USA) [29].

## Results

### Inter-experimental stability

**Figure 1:**
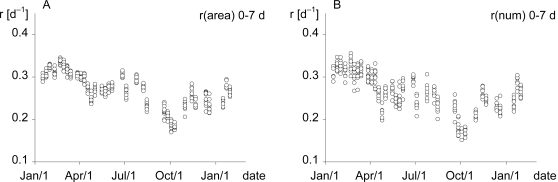
Growth rate variations of *Lemna gibba* over time. Variations in area-related (r_(area)_, A) and frond number-related (r_(num)_, B) average specific growth rate (day 0–7) of *Lemna gibba* for the time of investigation. Every replicate (beaker) is indicated by a dot.

The variability of the absolute growth rates over time is illustrated graphically, displaying the data from all ten beakers individually for all 35 experiments analysed (Fig. 1). The area-related average specific growth rate of *L. gibba* in individual beakers varied between 0.35 d^−1^ in early spring and 0.17 d^−1^ in autumn, whereas r_(num)_ varied between 0.36 d^−1^ and 0.15 d^−1^ respectively. In autumn the duckweed plants tended to have thicker fronds with a dark green colour. The variability between the ten replicates within each experiment was higher for the segmented growth rates compared to the average specific growth rates, especially at the beginning of the experiment (day 0–3) and for day 3–5 (data not shown).

**Figure 2:**
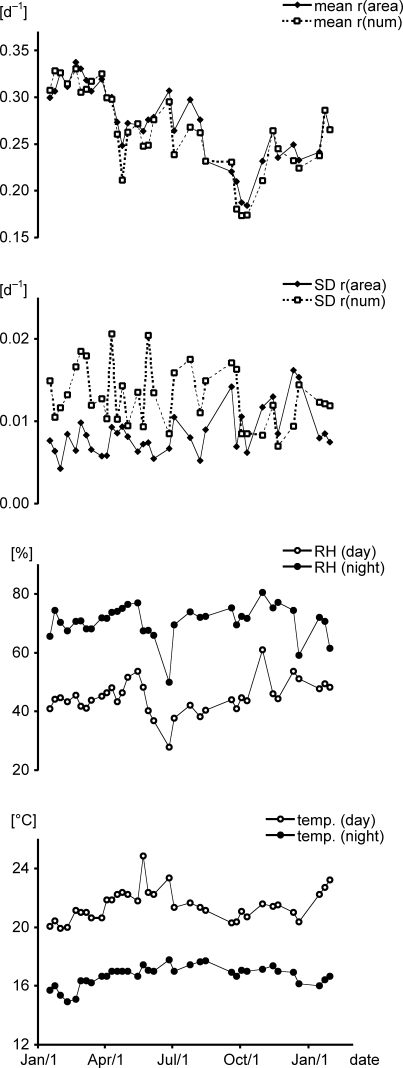
Comparison of duckweed growth rate and growth chamber conditions. Average specific growth rates (day 0–7) of *Lemna gibba* and conditions in the plant growth chamber over the time of investigation. Mean values and standard deviation (SD) of r_(area)_, r_(num)_, mean maximal temperature (temp., day and night) and mean maximal relative humidity (RH, day and night) are plotted against the date of the experiment.

Correspondingly, the mean average specific growth rates (mean of all 10 beakers) varied by a factor of about two: 0.34 d^−1^ for r_(area)_ and 0.33 d^−1^ for r_(num)_ in spring and 0.18 d^−1^ and 0.17 d^−1^ in autumn respectively, though laboratory conditions remained fairly constant and did not show a similar pattern over time (Fig. 2). Correlations of both average specific growth rates (r_(area)_ and r_(num)_) with day and night temperature and day and night relative humidity (RH) were calculated. Significant correlations were obtained with night temperature only (Spearman R = −0.418 for r_(area)_, p = 0.012; and R = −0.518 for r_(num)_, p = 0.001, respectively). Visual inspection of this correlation (Fig. 2) however reveals that the correlation is quite weak.

### Intra-experimental stability

Six full-size negative control experiments were performed and evaluated (n = 96 beakers for each experiment). The mean values of the average specific growth rates (r_(area)_ and r_(num)_) for all 16 groups of six replicates are shown in Table 1 and 2 (for every single systematic negative control experiment and for all six experiments combined). The mean growth rates of r_(area)_ and r_(num)_ were similar in single experiments, and for the average of all six water control experiments the mean growth rate of r_(area)_ and r_(num)_ was nearly identical (r = ∼0.267 d^−1^).The coefficient of variation (CV) for the average specific growth rates was 2.99% for r_(area)_ and 4.27% for r_(num)_, averaging all six systematic negative control experiments. In general, the statistical variation was higher for r_(num)_ compared to r_(area)_. This was also the case for all segmented growth rates (data not shown).

**Table 1:** Variability of the average specific growth rate based on frond area (r_(area)_).

r_(area)_	n	mean	mean CI		mean SD	mean SE	mean CV[%]	min CV[%]	max CV[%]
			−95%	+95%					
exp. 1	16×6	0.303	0.295	0.310	0.007	0.003	2.41	1.06	4.71
exp. 2	16×6	0.274	0.265	0.282	0.008	0.003	2.81	1.41	4.96
exp. 3	16×6	0.233	0.223	0.242	0.009	0.004	3.95	1.90	5.82
exp. 4	16×6	0.244	0.235	0.253	0.008	0.003	3.37	1.23	4.93
exp. 5	16×6	0.286	0.276	0.295	0.009	0.004	3.13	1.76	4.63
exp. 6	16×6	0.265	0.258	0.271	0.006	0.002	2.30	0.91	4.04
exp. 1–6	6×16×6	0.267	0.259	0.276	0.008	0.003	2.99±0.62		

Basic descriptive statistics of the area-related (r_(area)_) average specific growth rate (day 0–7) [d^−1^] of *Lemna gibba* for each systematic negative control experiment and for all six experiments combined, based on 16 groups of six randomly selected replicates. Data show the variability of the growth rate within and between experiments and document the stability of the experimental set-up.

**Table 2 pone-0003133-t002:** Variability of the average specific growth rate based on frond number (r_(num)_).

r_(num)_	n	mean	mean CI		mean SD	mean SE	mean CV[%]	min CV[%]	max CV[%]
			−95%	+95%					
exp. 1	16×6	0.301	0.290	0.312	0.011	0.004	3.57	1.80	5.91
exp. 2	16×6	0.247	0.234	0.259	0.012	0.005	4.85	1.67	9.17
exp. 3	16×6	0.243	0.231	0.255	0.012	0.005	4.78	2.84	7.28
exp. 4	16×6	0.240	0.230	0.250	0.010	0.004	4.09	1.85	6.70
exp. 5	16×6	0.293	0.279	0.308	0.014	0.006	4.71	2.53	6.14
exp. 6	16×6	0.272	0.261	0.282	0.010	0.004	3.63	1.29	5.19
exp. 1–6	6×16×6	0.266	0.254	0.278	0.011	0.005	4.27±0.58		

Basic descriptive statistics of the frond number-related (r_(num)_) average specific growth rate (day 0–7) [d^−1^] of *Lemna gibba* for each systematic negative control experiment and for all six experiments combined, based on 16 groups of six randomly selected replicates. Data show the variability of the growth rate within and between experiments and document the stability of the experimental set-up.

The variability of the growth rate calculated over section-by-section segmented growth rates (Table 3 and 4) reflects the stability of the growth rate within one single replicate over the three different time intervals. It was calculated for each single replicate. The mean coefficient of variation (CV) for groups of six controls was 7.16±2.97% for r_(area)_ and 8.58±2.56% for r_(num)_.

**Table 3 pone-0003133-t003:** Variability of the consecutive section-by-section segmented growth rate based on frond area.

r_(area)_	n	mean	mean SD	mean CV[%]	min CV[%]	max CV[%]
exp. 1	16×6	0.303	0.007	2.38	1.47	3.05
exp. 2	16×6	0.274	0.015	5.52	4.74	6.61
exp. 3	16×6	0.233	0.015	6.30	4.85	7.62
exp. 4	16×6	0.244	0.024	9.73	9.02	10.51
exp. 5	16×6	0.286	0.028	9.84	9.20	10.35
exp. 6	16×6	0.265	0.024	9.20	8.49	9.89
exp. 1–6	6×16×6			7.16±2.97		

Basic descriptive statistics for consecutive section-by-section segmented growth rate (0–3d, 3–5d and 5–7d) [d^−1^] of *Lemna gibba*: area-related (r_(area)_) mean coefficients of variation for single experiments expressing the stability of the growth rate for individual beakers in a group of six. In addition, mean and standard deviation of the coefficient of variation for all six experiments were calculated.

**Table 4:** Variability of the consecutive section-by-section segmented growth rate based on frond number.

r_(num)_	n	mean	mean SD	mean CV[%]	min CV[%]	max CV[%]
exp. 1	16×6	0.301	0.019	6.35	3.85	8.11
exp. 2	16×6	0.247	0.019	7.75	5.29	10.37
exp. 3	16×6	0.243	0.013	5.13	4.18	6.12
exp. 4	16×6	0.240	0.024	10.02	8.45	11.67
exp. 5	16×6	0.293	0.032	10.79	8.86	13.24
exp. 6	16×6	0.272	0.031	11.45	9.69	13.14
exp. 1–6	6×16×6			8.58±2.56		

Basic descriptive statistics for consecutive section-by-section segmented growth rate (0–3d, 3–5d and 5–7d) [d^−1^] of *Lemna gibba*: frond number-related (r_(num)_) mean coefficients of variation for single experiments expressing the stability of the growth rate for individual beakers in a group of six. In addition, mean and standard deviation of the coefficient of variation for all six experiments were calculated.

When comparing CVs of single experiments (Table 1–4) it is obvious that the variability of the test system did not necessarily increase with decreasing growth rate.

### Analysis of variance

Analysis of variance (ANOVA) of the six systematic negative control experiments simultaneously assessed inter- and intra-experimental stability. The statistical analysis of the effects of the independent variables (factors) experiment number, treatment and their interaction on both growth rates yielded highly significant effects for the experiment number on r_(area)_ and r_(num)_ due to the variation in absolute growth rates between the six experiments (Table 5). However, neither the factor treatment (here ‘pseudo-treatment’) nor the interaction between treatment and experiment number were statistically significant, indicating that no false positive results occurred in this experimental set-up.

**Table 5:** Analysis of variance of the systematic negative control experiments.

		0–7d	0–3d	3–5d	3–7d	5–7d
r_(area)_	exp. no.	**0.000**	**0.000**	**0.000**	**0.000**	**0.000**
	treatment	0.660	0.258	0.970	0.986	0.419
	interaction	0.131	0.209	0.212	0.099	0.649
r_(num)_	exp. no.	**0.000**	**0.000**	**0.000**	**0.000**	**0.000**
	treatment	0.288	0.332	0.258	0.662	0.365
	interaction	0.576	0.654	0.340	0.133	0.313

Statistical analysis of the systematic negative control experiments: p-values were calculated by analysis of variance (ANOVA, F-test) for the average specific growth rates and segmented growth rates of *Lemna gibba*. Effect of experiment number (exp. no.), treatment and their interaction on the growth parameters r_(area)_ and r_(num)_ from six independent systematic negative control experiments. Significant values (p<0.05) are bold.

## Discussion

In this study the observed changes in area-related and frond number-related growth rates over about one year did not correlate well with changes of temperature and RH in the growth chamber (Fig. 2). Since even minor changes in the *L. gibba* bioassay procedures (like test vessel material, sterilisation and axenic culturing procedures) influence the sensitivity of the bioassay [30], our study maintained the selected growth conditions in the laboratory, the materials used and the handling procedures as constant as possible over the whole period of investigation. Thus the observed variations in growth rates over time might be caused by different seasonal conditions and endogenous periodicities of *L. gibba*.

The phenomenon of seasonally altered duckweed growth (under constant laboratory conditions) has also been observed elsewhere. Based on the number of fronds Wang [24] suggested a seasonal variation in the growth of *L. minor*. Pirson et al. [31] described annual rhythmic changes in root growth of *L. minor* under controlled conditions, while Bornkamm [32] reported for the same duckweed species seasonal changes in the rate of dry matter production and the protein/carbohydrate ratio, which seemed to correspond to periodic changes of a field population. However, both authors observed minimal growth of *L. minor* in winter months, while in our study the lowest growth rates occurred in autumn associated with changes in the appearance of the fronds of *L. gibba*. Indications of an annual growth cycle and periodical changes in frond morphology of *L. gibba* were reported by Tillberg et al. [23] with small, light colonies with fast fresh weight-related growth rate occurring during the summer months and larger, heavier colonies which grew more slowly during the rest of the year. The two growth forms also differed in sensitivity to treatment with the growth regulators abscisic acid and 6-benzyl-aminopurine: heavy plants seemed to be more sensitive.

Given the fact that the absolute growth rates may vary by a factor of two in the course of a year, detailed investigations are needed to determine a possible relationship between absolute growth rates and the ecotoxicological sensitivity of *Lemna* spp. to toxic substances with different modes of action. Thus, additional data are necessary to decide whether the standardised minimum growth rate of r = 0.275 d^−1^ is a sufficient validity criterion for all kinds of substances in *Lemna* bioassays.

We furthermore compared the CVs of the different growth rates and calculation parameters. Both growth parameters determined in this study yielded similar average specific growth rates, but r_(area)_ had always lower CV values than r_(num)_. This is most probably due to the fact that the area of fronds is a continuous variable, whilst the number of fronds increases discontinuously. Thus r_(area)_ seems to be a more stable parameter to measure the growth rate, whilst r_(num)_ remains important as basic parameter which is always accessible. These results confirm the findings of Cedergreen et al. [33] who reported the area-related relative growth rate to be the most precise non-destructive calculation parameter.

In our investigations, both average specific growth rates (r_(area)_ and r_(num)_) measured did not always meet the single validity criterion of the test guidelines (0.275 d^−1^ [21], [22] or 0.230 d^−1^ [20], corresponding to an approximately seven- or five-fold increase, respectively). This deviation is likely to be due to the light-dark regime of illumination used in this study and the consequent daily alteration in temperature. This regime was used since it better reflects the natural physiological conditions of duckweed growth.

In order to empirically assess the hypothesis of altered sensitivity at different growth rates, evidence has to be provided that the used experimental set-up is stable, i.e. the experimental conditions do ensure a low variability within and between experiments, even at low growth rates. Therefore, two new validity criteria are proposed.

In our study, the observed CVs of both area-related and frond number-related average specific growth rates (2.99% and 4.27%, respectively) were small, indicating a good stability of the entire experimental set-up over the entire period of time, even at low growth rates. The values are in the same order of magnitude as those measured for six control replicates in an ISO *Lemna minor* ringtest, with CVs of average specific growth rates of 3.68±2.65% (n = 32 tests) for r_(area)_ and 4.19±2.48% (n = 68 tests) for r_(num)_ (in that analysis only valid tests with an average specific growth rate of r≥0.275 d^−1^ were included; M. Eberius, LemnaTec, Würselen, Germany, personal communication). It is therefore proposed that a maximum CV of the average specific growth rate between control replicates of 10% may be another useful and not too stringent validity criterion for *L. gibba* in duckweed bioassays. A CV-value of 10% has already been included in the ISO guidelines, however only as a recommendation for a desirable good systematic negative control experiment (non-toxicant test) with new test facilities [21].

A further validity criterion already applied for the freshwater algae and cyanobacteria growth inhibition test [28] is the variation of growth within each control replicate, calculated as the mean CV for section-by-section segmented growth rates in the control cultures, which must not exceed 35%. For the *Lemna* bioassays no such validity criterion exists so far due to lack of a broad database. On the basis of our data we propose as a further useful validity criterion a maximum CV of 20% for within replicate variation for *L. gibba* tests. Such a criterion seems to be appropriate in order to examine whether the control growth rate remains constant or if it varies either due to an initial lag phase or due to nutrient restrictions or overcrowding at the end of the experiment.

Both proposed complementary validity criteria should be confirmed for other *Lemna* spp. in similar investigations. In addition, possible relationships between the inter- and intra-experimental stability and the ecotoxicological sensitivity of *Lemna* spp. to toxic substances with different modes of action should be determined.

Additional information about the stability of an experimental set-up can be obtained by evaluation of statistical significance based on analysis of variance F tests of data from several systematic negative control experiments. With this type of analysis false positive results (that may occur due to uncontrolled variations within the experimental set-up) can be excluded with high certainty, if there is neither a significant ‘pseudo-treatment’ effect nor an interaction of ‘pseudo-treatment’ and experiment number. To document low variability of the test system may be of special importance, when low concentrations are to be tested, e.g. mixtures of single test substances which alone have no significant concentration effect [34].
